# Visualizing non-equilibrium lithiation of spinel oxide via *in situ* transmission electron microscopy

**DOI:** 10.1038/ncomms11441

**Published:** 2016-05-09

**Authors:** Kai He, Sen Zhang, Jing Li, Xiqian Yu, Qingping Meng, Yizhou Zhu, Enyuan Hu, Ke Sun, Hongseok Yun, Xiao-Qing Yang, Yimei Zhu, Hong Gan, Yifei Mo, Eric A. Stach, Christopher B. Murray, Dong Su

**Affiliations:** 1 Brookhaven National Laboratory, Upton, New York 11973, USA; 2 Department of Chemistry, University of Pennsylvania, Philadelphia, Pennsylvania 19104, USA; 3 Department of Materials Science and Engineering, University of Maryland, College Park, Maryland 20742, USA

## Abstract

Spinel transition metal oxides are important electrode materials for lithium-ion batteries, whose lithiation undergoes a two-step reaction, whereby intercalation and conversion occur in a sequential manner. These two reactions are known to have distinct reaction dynamics, but it is unclear how their kinetics affects the overall electrochemical response. Here we explore the lithiation of nanosized magnetite by employing a strain-sensitive, bright-field scanning transmission electron microscopy approach. This method allows direct, real-time, high-resolution visualization of how lithiation proceeds along specific reaction pathways. We find that the initial intercalation process follows a two-phase reaction sequence, whereas further lithiation leads to the coexistence of three distinct phases within single nanoparticles, which has not been previously reported to the best of our knowledge. We use phase-field theory to model and describe these non-equilibrium reaction pathways, and to directly correlate the observed phase evolution with the battery's discharge performance.

## Abstract (web summary)

Non-equilibrium intercalation reactions may determine the performance of lithium-ion battery materials undergoing lithiation, but it is difficult to probe in real time. Here, the authors use *in situ* electron microscopy to identify kinetically-driven phase evolution in magnetite single nanoparticles.

## Graphical abstract

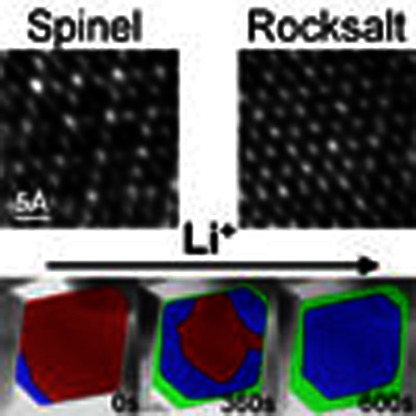

The spinel transition metal oxide family—which includes LiMn_2_O_4_, Li_4_Ti_5_O_12_, Fe_3_O_4_, Co_3_O_4_, Mn_3_O_4_, among others—is an important group of compounds, and one that sees considerable use as electrode materials in lithium-ion batteries^1^,^2^,^3^,^4^,^5^,^6^,^7^. Specifically, magnetite (Fe_3_O_4_) is a promising candidate as an anode material, as it is both inexpensive and non-toxic. Importantly, it also has a high electronic conductivity and can store up to eight Li ions per formula unit, which leads to a high theoretical specific capacity of 926 mAh g^−1^ (refs 8, 9, 10, 11, 12, 13, 14, 15, 16, 17, 18, 19, 20). The structure of Fe_3_O_4_ is inverse spinel ((Fe^3+^)_8a_[Fe^2+^Fe^3+^]_16d_O_4_, denoted by Wyckoff notation), where there are 64 tetrahedral sites in a unit cell (one-eighth of them are 8a sites occupied by 8 Fe^3+^ cations) and 32 octahedral sites (half of them are 16d sites occupied by 8 Fe^2+^ and 8 Fe^3+^ cations), leaving behind 56 empty tetrahedral (8b and 48f) sites and 16 empty octahedral (16c) sites in the interstitial space to allow for accommodation of guest Li^+^ ions^17^,^18^,^19^,^20^. The lithiation of spinel iron oxide was investigated by Thackeray *et al*.^16^,^17^ using a combination of open-circuit voltage (OCV) measurements and X-ray diffraction, and they posited that the overall lithiation of Fe_3_O_4_ follows the reaction equation shown in equation (1):


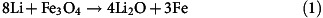


They suggested that during the initial stage of lithium insertion, the Fe^3+^ cations at tetragonal 8a sites are distorted into the nearby octahedral 16c sites by inserting Li^+^ ions into the octahedral 16c sites, which leads to a lattice reconstruction of the spinel structure to form the rocksalt structure, that is, (Li^+^_*x*_Fe^3+^)_16c_[Fe^2+^Fe^3+^]_16d_O_4_ (refs 16, 17, 18, 19, 20, 21). This is an intercalation process, and thus it does not lead to a significant volume change. They proposed that further lithiation triggers destruction of the rocksalt structure along with an extrusion of metallic Fe from the material, which is commonly known as a conversion reaction. During the conversion reaction, the rocksalt structure transforms into a composite which is composed of both metallic Fe and Li_2_O. Critically, this conversion leads to a drastic volume expansion^9^,^10^,^11^,^12^,^13^,^14^,^15^.

Fe_3_O_4_ shows dramatic differences in electrochemical performance as a function of discharge rate (‘C rate'), with the intercalation process not readily observed in the discharge voltage profiles^9^,^10^,^11^. These differences cannot be understood from simple consideration of the reaction equation, which describes how these processes occur in an equilibrium condition. In one related example, the positive electrode material LiFePO_4_ (LFP) exhibits a solid-solution transformation at high rates when undergoing an intercalation reaction, specifically when the two-phase transition process is suppressed above a certain overpotential^22^,^23^. Thus, it is suggested that for Fe_3_O_4_ the overall charge-discharge performance depends strongly on the kinetics of specific discharge mechanisms. This in turn determines the realistic redox reaction characteristics that the battery sees in working conditions.

Finally, it is well-known that the rate capability of electrodes can be improved by reducing the material size from the micrometre to the nanometre regime. This improvement is due to both the increase in surface area and the reduction of electron/ion diffusion length^24^,^25^,^26^. However, in the case of nanoscale electrodes, the overall kinetics of lithiation is further complicated by local inhomogeneities that can occur in the electrochemical conditions. Besides the presence of both intercalation and conversion reactions, all of these facts underscore the importance of understanding the phase transitions of Fe_3_O_4_ that occur in non-equilibrium conditions, as it is these parameters that must be optimized when considering spinel-based electrode materials in electrochemical applications^22^.

In this work, we investigate how non-equilibrium intercalation and conversion reactions proceed by studying the lithiation process in real time. X-ray scattering or Mössbauer spectroscopy is most often used to identify the phase transformations that occur in nanosized electrode materials^16^,^19^, but these techniques have the disadvantage of providing only average information, and generally they lack the ability to probe reaction kinetics in real time. Critically, it is important to undertake lithiation studies *in situ*, as *ex situ* studies may provide inaccurate information due to changes that may occur during removal of the electrode materials from an actively lithiated condition^22^,^27^. In contrast, transmission electron microscopy (TEM) allows direct, real-time information to be obtained from structures at the atomic scale. The need for *in situ* observations of this type has been increasingly recognized, and thus there have been many recent reports of the use of *in situ* TEM techniques to study alloying and conversion reactions^28^,^29^,^30^,^31^,^32^,^33^,^34^,^35^,^36^,^37^,^38^. However, directly visualizing phase transitions that occur during intercalation reactions is challenging because the volume change that occurs during this type of reaction is negligible^39^,^40^,^41^,^42^. This prevents the use of standard diffraction contrast imaging techniques. In this work, we utilize a strain-sensitive, bright-field scanning TEM (BF-STEM) imaging technique to overcome this challenge. This approach allows us to observe the phase changes that occur in monodisperse Fe_3_O_4_ nanoparticles *in situ* during the intercalation reaction, and follow the subsequent conversion reaction directly. By integrating *ex situ* synchrotron X-ray and *in situ* TEM approaches, we capture in detail the mechanisms by which lithiation occurs and relate this directly to the reaction kinetics. Furthermore, we support these observations with both density functional theory (DFT) calculations and non-equilibrium phase-field theory to describe the atomistic processes from first principles and phenomenological perspective, respectively. This work highlights the importance of exploiting advanced TEM techniques to reveal reaction inhomogeneities at the nanoscale.

## Results

### Material structures and electrochemical properties

**Figure 1:**
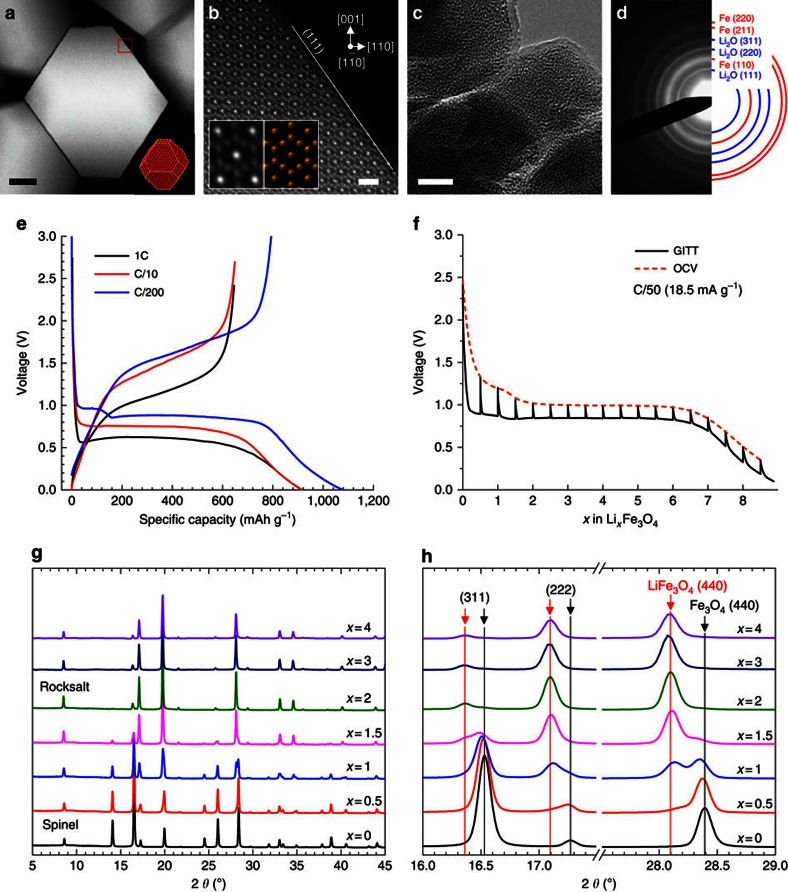
Electrode materials and electrochemical performance. (**a**) STEM image of the pristine Fe_3_O_4_ single crystals. Inset showing the crystals in the truncated octahedral shape. (**b**) High-resolution HAADF-STEM image showing spinel structure along [1–10] zone axis. Inset showing enlarged STEM image compared with the atom model. (**c**) TEM image and (**d**) SAED pattern of the fully lithiated electrode, confirming the nanocomposite of Fe nanocrystals in amorphous Li_2_O matrix. Scale bars, (**a**,**c**) 20 nm; (**b**) 1 nm. (**e**) First-cycle charge/discharge profiles of coin-cell batteries at rates of 1C, C/10 and C/200 (that is, 926 mA g^−1^, 92.6 mA g^−1^ and 4.63 mA g^−1^). (**f**) GITT and OCV profiles in the first discharge cycle measured at C/50 (18.5 mA g^−1^). GITT measurements were performed by applying an intermittent current of C/50 for 3.125 h followed by a 24 h relaxation period. (**g**) SXRD patterns of Li_*x*_Fe_3_O_4_ at various SOCs (*x*=0, 0.5, 1, 1.5, 2, 3, 4). (**h**) Enlarged SXRD patterns showing detailed peak shifts of (311), (222) and (440) reflections. Fe_3_O_4_ and LiFe_3_O_4_ phases are marked by black and red arrows. X-ray wavelength is 0.72768 Å.

The as-synthesized Fe_3_O_4_ nanoparticles are *ca.* 80 nm truncated octahedrons, as shown in Fig. 1a and Supplementary Fig. 1. Atomic-resolution high-angle annular dark-field (HAADF) STEM images (as in Fig. 1b) indicate that the pristine Fe_3_O_4_ nanoparticles are single-crystalline with the inverse spinel structure (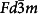 space group) and terminated with {111} crystal planes. To characterize the electrochemical performance, voltage profiles at different C-rates (1C, C/10 and C/200, or 926 mA g^−1^, 92.6 mA g^−1^ and 4.63 mA g^−1^, respectively) are shown in Fig. 1e. The first discharge capacity is 1,080 mAh g^−1^ at C/200, and it decreases to 916 mAh g^−1^ and 914 mAh g^−1^ for C/10 and 1C, respectively, close to the theoretical capacity of 926 mAh g^−1^. The capacity excess in the initial discharge is generally believed due to side reactions involving electrolyte decomposition and formation of solid electrolyte interphases^43^,^44^,^45^, while the following cyclic tests show a rate performance (Supplementary Fig. 2) that is comparable to previous reports^10^,^11^,^12^. After full discharge (lithiation), the electrode materials have changed their structure and morphology dramatically to form a nanocomposite consisting of ultrafine (<5 nm) Fe nanoparticles and an amorphous Li_2_O matrix phase (Fig. 1c), as confirmed by electron diffraction (Fig. 1d). It is noted that two distinct plateaus appear in the C/200 discharge curve, corresponding to the intercalation reaction and conversion reaction, respectively^13^,^14^,^15^,^17^, whereas the intercalation plateau is absent from the relatively faster discharge curves at rates of 1C and C/10. This can be due to enhancements in the reaction kinetics that can occur at high discharge current densities (such as voltage polarization). To justify this hypothesis, we employed the galvanostatic intermittent titration technique (GITT)^46^ for a discharge cycle at C/10 to measure the OCV profile: this reflects the equilibrium redox potentials at different states of charge (SOCs). Figure 1f clearly indicates the voltage polarization between the OCV and regular discharge profile, and confirms the existence of an intercalation plateau which is related to the charge rate. The intercalation process can generally be expressed as equation (2):


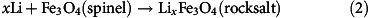


where *x* is the lithium content per formula. For an intercalation reaction, *x* is supposedly between 0 and 2 (refs 16, 17, 18), and its upper limit depends on the total amount of lithium ions accommodated at the tetragonal 8a sites^16^,^17^. Further lithiation would trigger the conversion reaction. This is also confirmed by high-resolution TEM (HRTEM) imaging of *ex situ* lithiated Li_*x*_Fe_3_O_4_ with Li content *x*=2, 3, 4, 5, 6, 7, 8 (Supplementary Fig. 3). These HRTEM images and selected area electron diffraction (SAED) patterns show the transition from rocksalt Li_*x*_Fe_3_O_4_ to Fe+Li_2_O composite in a gradually proceeding manner. Since the intercalation reaction only involves Li content *x*<2, it is crucial to focus our study on the first half of the lithiation. To study the intercalation process further, we utilized synchrotron X-ray diffraction (SXRD) to identify the phase changes at various SOCs of *x*=0, 0.5, 1, 1.5, 2, 3 and 4, as shown in Fig. 1g,h. The series of SXRD patterns show that the electrode materials transfer from spinel structure to rocksalt structure with increasing Li composition (detailed indexing is presented in Supplementary Fig. 4). From the enlarged patterns in Fig. 1h, we indeed identified the two stable Fe_3_O_4_ and LiFe_3_O_4_ phases. The nanosized Fe phase that resulted from the conversion reaction cannot be captured by SXRD even up to *x*=4; however, its presence was confirmed using TEM (Supplementary Fig. 5). These observations clearly indicate that SXRD—due to its lack of sensitivity in detecting nanoscale particles, and its averaging of the overall phase information—is not able to precisely determine either the initiation of the conversion reaction or to follow its evolution. In addition, the absence of an intercalation plateau in the cycling profiles of 1C and C/10 implies that the intercalation process is bypassed at high rates. Although the SXRD results clearly show the existence of a rocksalt intermediate phase, which arises from the intercalation reaction, the presence of this phase might also be due to a possible structure relaxation and phase separation that occurs post-mortem. Effects of this type have been reported in LFP system, where *ex situ* characterization is shown to inaccurately describe the reaction processes that are occurring^21^. In addition, the intercalation phase transition can either follow a two-phase model or solid-solution process depending on the details of the reaction kinetics, a process which cannot be resolved from the *ex situ* study. To solve these issues, we have utilized *in situ* electron microscopy approaches to determine which reaction pathways occur in spinel oxide.

### Phase evolution by *in situ* electron diffraction

**Figure 2:**
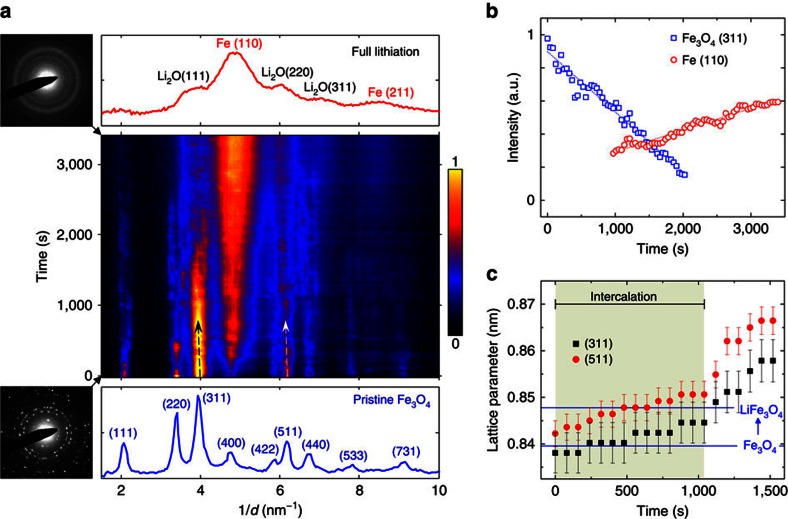
Phase evolution probed by *in situ* electron diffraction. (**a**) Electron diffraction intensity profile (the colour map in the middle) as a function of reaction time during an *in situ* lithiation of Fe_3_O_4_ nanoparticles (see Supplementary Movie 1). The SAED patterns and corresponding radially integrated intensity profiles obtained at pristine (0 s) and fully lithiated (3400, s) states are shown below and above the colour map, respectively. Arrows indicate diffraction peak shift towards a lower value in the reciprocal space. The sample was lithiated at a rate of ∼980 mA g^−1^. (**b**) Intensity profiles of Fe_3_O_4_ (311) and Fe (110) Bragg reflections during the lithiation, indicating the phase evolution of Fe_3_O_4_ and Fe. (**c**) Lattice parameter measured from Fe_3_O_4_ (311) and (511) reflections showing an incremental shift as a function of lithiation time, which quantitatively agrees with the SXRD results. Error bars are given by 1 pixel precision of SAED measurements. Blue lines indicate lattice parameters of Fe_3_O_4_ and LiFe_3_O_4_ measured by SXRD in Fig. 1g.

We utilized a dry cell set-up for our *in situ* TEM investigation^36^,^37^,^38^. This approach can precisely track the phase evolution at very high spatial resolution. Since there are no liquid organic electrolyte involved in the reactions, to verify the consistency in the phase transitions between the *ex situ* and *in situ* experiments, we tracked the dynamical phase evolution using *in situ* electron diffraction throughout the entire lithiation process, as shown in Fig. 2a and Supplementary Movie 1 The radially integrated intensity profiles from a series of time-sequenced SAED patterns are plotted as a function of lithiation time, with the intensity represented by false colours (Fig. 2a). It is obvious that both the position and intensity of the Bragg reflections change as the lithiation proceeds, verifying the overall phase transformation from pristine Fe_3_O_4_ to the eventual Fe and Li_2_O composite (and in the same manner as the *ex situ* results). Using the intensity profiles of Fe_3_O_4_ (311) and Fe (110) SAED peaks as a measure, the gradual evolution of the phase transformation becomes clear, as depicted in Fig. 2b. It is also worth noting that the Bragg peaks of the Fe_3_O_4_ phase display a negative shift in reciprocal space (as indicated by arrows in Fig. 2a) during the initial stage of lithiation (up to ∼1,000 s), which corresponds to an increase in the lattice parameter as Li^+^ ions are inserted into the Fe_3_O_4_ lattice (Fig. 2c). This lattice parameter increase is quantitatively consistent with the phase transition from Fe_3_O_4_ to LiFe_3_O_4_, as extracted from our SXRD measurements. After ∼1,000 s, the lattice expansion becomes more severe, indicating that the subsequent lithiation was dominated by the conversion reaction, as per equation (3).


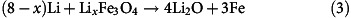


### Visualization of two-step lithiation by *in situ* BF-STEM

**Figure 3:**
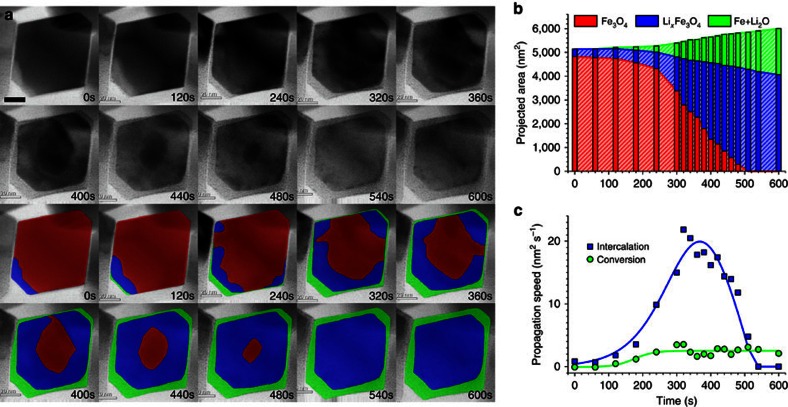
*In situ* observation of two-step phase transformation during lithiation. (**a**) BF-STEM image series showing phase evolution during lithiation. The overlaid false colours indicate different phases: pristine Fe_3_O_4_ (red), Li-inserted Li_*x*_Fe_3_O_4_ (blue) and Fe+Li_2_O composite after conversion (green). Scale bar, 20 nm. The sample was lithiated at a rate of ∼2,700 mA g^−1^. (**b**) Projected areas of the three phases as a function of time. (**c**) Propagation speed of intercalation and conversion as a function of time.

After verifying that the phase transformation occurs using *in situ* SAED, we performed an *in situ* BF-STEM observation to follow how the structure evolves during the entire lithiation process in real space, as shown in Fig. 3a and Supplementary Movie 2. The time-sequenced BF-STEM images record the intensity of the transmitted electrons scattered to lower angles, as well as the direct beam (involving the most coherent electron scattering). This signal is strain sensitive and thus enables direct visualization of the small lattice changes that occur during the intercalation process (Supplementary Fig. 6)^47^. We show that this is essential observing intercalation reactions in real time. This is in contrast to other, more conventional imaging techniques (such as HAADF-STEM or BF/DF TEM) which do not have the requisite image sensitivity to directly observe this process (sensitivity to strain is not satisfied due to incoherent contrast or bending contour). The first intercalation step follows the reaction described in equation (2) to generate the lithium-inserted Li_*x*_Fe_3_O_4_ phase (shown with a lighter contrast). These regions will be further lithiated in the subsequent conversion reaction, which is also accompanied by the extrusion of ultrafine metallic Fe nanoparticles to form a composite with the amorphous Li_2_O. For better visualization, we use false colours to distinguish the pristine Fe_3_O_4_ (red), Li-inserted Fe_3_O_4_ (blue) and completely conversion composite (green) phases and their evolution as a function of time. This was also verified using electron energy-loss spectroscopy (EELS) in STEM (Supplementary Fig. 7). We note that, under realistic electrochemical environment, the lithiation process shows a non-equilibrium reaction pathway, that is, the conversion reaction starts to take place before the intercalation completely finishes. This may be likely due to the fact that the diffusion of lithium on the Fe_3_O_4_ particle surface is faster than that through the lithiated Li_*x*_Fe_3_O_4_ phase, which can cause a sufficiently large lithium concentration at the particle surface and thereby trigger the conversion reaction to happen at an early stage (but still after the intercalation reaction). It is obvious that both intercalation and conversion reactions follow a ‘shrinking-core' mode, proceeding from the outer surface to the inner region^37^. Specifically, the initial intercalation trajectory does not exhibit any preferential directions, in accord with the zigzag Li^+^ diffusion paths in three-dimensional (3D) tunnels^16^, whereas the subsequent conversion tends to propagate through the outer facets, that is, {111} planes. As an approximate measure of reaction kinetics, we quantified the projected areas of the three phases versus the lithiation time, displayed in Fig. 3b. This figure shows that the propagation speed of the intercalation process is about 1 order of magnitude faster than the following conversion reaction (Fig. 3c). We note that these measurements are from one single particle, where the electrochemical conditions are supposed to be identical. This is consistent with the fact that full conversion needs multiple Li-ion transfers, when compared with the intercalation process. We do not believe this to be an effect resulting from local electrochemical inhomogeneity.

### HRTEM characterization

**Figure 4:**
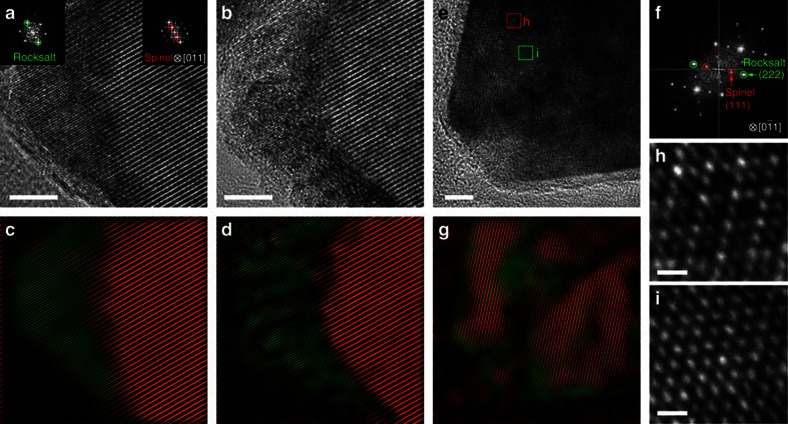
Atomic structural evolution of phase transformation. (**a**,**b**) HRTEM images of a Fe_3_O_4_ single crystal showing the spinel and rocksalt phases during *in situ* lithium intercalation. Insets showing the FFT of the spinel (top-right of panel) and rocksalt (top-left of panel) structures along [0–11] zone axis. (**e**) HRTEM image of a partially lithiated Fe_3_O_4_ single crystal from *ex situ* lithiation. (**f**) The FFT of **e**. (**h**,**i**) The enlarged HRTEM images of boxed regions in **e** showing spinel and rocksalt structures, respectively. (**c**,**d**,**g**) Filtered images of (**a**,**b**,**e**) using two sets of spinel (red) and rocksalt (green) diffraction spots to indicate the corresponding phase distribution. The samples were lithiated at ∼150 mA g^−1^ for *in situ* lithiation, and 92.6 mA g^−1^ for *ex situ* lithiation. Scale bars: (**a**,**b**,**e**) 5 nm; (**h**,**i**) 5 Å.

For a fundamental understanding of the intercalated phase, we conducted HRTEM imaging to track the atomic structure changes for both *in situ* and *ex situ* scenarios, as shown in Fig. 4. A new phase with the rocksalt structure was observed *in situ* as Li^+^ ions were inserted into the spinel Fe_3_O_4_ phase (Fig. 4a,b). Using two sets of diffraction spots from the fast Fourier transform (FFT, Fig. 4a insets), we can separately map the distribution of the spinel (red) and rocksalt (green) phases and thus visualize the propagation of the intercalation front in the reacting Fe_3_O_4_ crystal (Fig. 4c,d). Similarly, in an *ex situ* lithiated sample that was discharged to 0.9 V, we reproduced this result, and found a similar mixture of spinel and rocksalt phases (Fig. 4e,g). In the pristine spinel Fe_3_O_4_ crystals, Fe cations occupy the tetrahedral 8a sites and octahedral 16d sites, which give the uneven TEM contrast (dim at the diamond centre) shown in Fig. 4h. Upon lithium insertion, all the cations migrate to the octahedral sites (16c and 16d), leading to a uniform contrast (Fig. 4i). This means that the inserted Li ions occupy the 16c sites and also repel the adjacent Fe cations from the 8a to the 16c sites to form Li_*x*_Fe_3_O_4_ crystals with the rocksalt structure, with lithium composition up to *x*=1. It is also noted from previous reports that the excess Li ions (up to *x*=2) could possibly insert into the 8b and 48f sites to form Li_2_Fe_3_O_4_ phase without Fe extrusion^16^,^17^. However, this particular phase was not experimentally identified from TEM observations. Nevertheless, the intercalation process goes through a two-phase mode and forms an intermediate phase of Li_*x*_Fe_3_O_4_ (refs 16, 17, 18). Thereafter, the conversion reaction occurs and the Li_*x*_Fe_3_O_4_ phase is decomposed into Li_2_O and metallic Fe nanocrystals, which can be observed as the speckles in Fig. 4b,d. Combining both *in situ* TEM imaging and *ex situ* SXRD results, we now can conclude that the intercalation proceeds in a two-phase ‘nucleation and growth' manner, resulting in sharp interfacial boundaries between the spinel and rocksalt phases, as expressed in equation (2).

### First principles calculations and phase-field modelling

**Figure 5:**
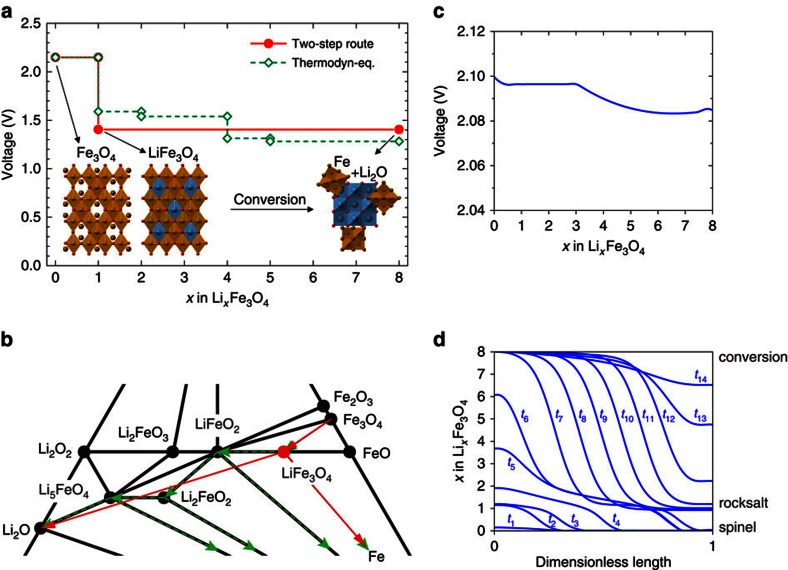
DFT calculation and phase-field simulation of lithiation process in Fe_3_O_4_ crystals. (**a**) The voltage profiles as a function of Li composition (*x*=0–8) calculated by DFT showing both thermodynamic equilibrium (green dashed lines) and two-step reaction (red solid lines) routes for an entire lithiation process. The inset models showing crystal structures of Fe_3_O_4_ and LiFe_3_O_4_ phases during the intercalation process and a schematic of Li_2_O plus metallic Fe after the complete conversion reaction. Colour spheres representing Fe (yellow), Li (blue) and O (red). (**b**) Li–Fe–O phase diagram with overlaid arrow lines showing lithiation pathways through thermodynamic equilibrium multistep route (green dashed lines) or experimentally observed two-step route (red solid lines). (**c**) Discharge voltage profiles calculated from the phase-field modelling. (**d**) Li composition profiles along one-dimensional length as a function of reaction time simulated by the phase-field theory (also see Supplementary Movie 3). The time intervals are uniform between adjacent time stamps.

DFT computation has been performed to calculate the voltage profile of the lithiation process under the thermodynamic equilibrium condition^48^,^49^,^50^,^51^,^52^. The calculated reaction pathway has multiple voltage plateaus (green dashed curve in Fig. 5a) with intermediate phases, such as LiFeO_2_, Li_2_FeO_2_ and Li_5_FeO_4_ (Fig. 5b). Given that the LiFe_3_O_4_ is an intermediate phase experimentally observed from SXRD, our DFT calculations also considered a two-step reaction route (red curve in Fig. 5a), including the intercalation plateau (Li+Fe_3_O_4_→LiFe_3_O_4_) and the conversion plateau (LiFe_3_O_4_→Li_2_O+Fe). The ground-state LiFe_3_O_4_ structure as illustrated in the inset crystal models of Fig. 5a possesses a similar structural framework (that is, [Fe]_16d_O_2_ octahedron) that was inherited from the spinel Fe_3_O_4_ structure. Consistent with the ground-state LiFe_3_O_4_ structure calculated in the DFT computation, Li ions insert into the octahedral 16c openings, and repel the nearby 8a Fe ions to the other surface-sharing 16c site, which forms the structure identical to that observed in the HRTEM images in Fig. 4. In addition, since the octahedral 16c sites are interconnected as a 3D channel, the initial intercalation would proceed in a disordered pattern, which explains the irregular patterns that are observed during the shrinking-core process in Fig. 3. After all of the octahedral 16c sites are filled by Li, the rocksalt lattice becomes more isotropic to the incoming Li ions, which makes the subsequent conversion most likely to propagate across the outline of crystal, again as observed in Fig. 3.

## Discussion

Given the basics of chemical thermodynamics, the equilibrium lithiation should go through the LiFe_3_O_4_ phase and the subsequent Fe+Li_2_O composite in two non-overlapping processes, as proposed by Thackeray *et al*.^16^,^17^,^18^. However, our *in situ* results show a clear overlap between the two steps, suggesting that the kinetic effects play an important role during lithiation. On the other hand, the phase-field theory, taking into account the effect of overpotentials, has succeeded in explaining the electrochemical kinetics in lithium-ion battery systems^53^,^54^,^55^. We performed the phase-field simulation in the frame of non-equilibrium thermodynamics based on the Butler–Volmer equation^55^,^56^ and the Cahn–Hilliard equation^23^,^57^. To simulate the two-step reaction, we constructed a homogeneous free-energy function with three local minima corresponding to the pristine, intercalation and conversion phases, respectively. In addition, the kinetic contributions from overpotential and volumetric strain have also been included (details in Supplementary Methods). The calculated discharge voltage profile is shown in Fig. 5c, which qualitatively agrees with the experimental curves in Fig. 1e. Figure 5d shows the calculated lithium compositional profiles as a function of time (*x*-axis is the dimensionless lithiation direction and *y*-axis is the Li composition, details shown in Supplementary Movie 3). It is found that the formation of LiFe_3_O_4_ phase is predominant in the early stage of lithiation, whereas the conversion reaction initiates immediately afterward and propagates before the complete intercalation, resulting in a mixed lithiation behaviour, which is in good agreement with the *in situ* STEM observation (Fig. 3a). It is well-known that the lithiation process (redox stage and reaction speed) in a real battery is heterogeneous due to the fluctuation of local environments^22^. Here we reasonably assume that the local electrochemical conditions are identical for these two reactions within one nanoparticle. By comparing the *in situ* STEM results with phase-field simulation, we believe that the coexistence of Fe_3_O_4_, Li_*x*_Fe_3_O_4_, and Fe+Li_2_O phases are due to the competition between the intercalation and conversion reactions. The overall electrochemical kinetics dictates to the applied C-rate, which in turn determines the propagation speeds of the interfaces of Fe_3_O_4_/Li_*x*_Fe_3_O_4_ and Li_*x*_Fe_3_O_4_/Fe+Li_2_O. Although the reaction speed of intercalation is much faster, the conversion reaction accommodates more Li ions. Therefore, in terms of total lithium insertion capacity, both reactions give important contributions to the overall energy storage rate.

The kinetic lithiation mechanism we proposed here can accommodate the following phenomena which could not be interpreted by the equilibrium theory. (1) As for the discharge profiles in Fig. 1e and in literature^9^,^10^,^11^, even the intercalation reaction has not completed, the occurrence of conversion reaction on the surface will reduce the apparent discharge voltage, which consequently flattens the first plateau. (2) As for the previous debate on the existence of metallic Fe extrusion^16^,^17^,^18^,^19^, due to the kinetic effect, the conversion reaction can happen at the same time of the intercalation reaction, which induces the formation of metallic Fe. For the discharge process at a low rate, the extrusion of metallic Fe is not expected to be observed.

In summary, we have investigated the lithiation mechanism of spinel magnetite using an *in situ* strain-sensitive, BF-STEM technique, as well as DFT computation and phase-field simulation. By explicit visualization of the two-step intercalation-conversion process of the lithiation in Fe_3_O_4_ nanocrystals, we found that the initial lithium intercalation leads to formation of the rocksalt LiFe_3_O_4_ phase in a two-phase reaction mode, and the lithium intercalation process significantly overlaps with the subsequent conversion reaction within a single nanoparticle, leading to indistinctness of the discharge profiles. This scenario aslo clarifies the mechanism of metallic Fe extrusion during the intercalation process. Our findings have elucidated the ionic occupancy on the atomic level and revealed how rate-dependent kinetic effects can affect the reaction pathway at the single-particle scale. These findings highlight the importance of advanced *in situ* electron microscopy techniques in the field of lithium-ion batteries and provide valuable insights to improve the electrochemical performances of other spinel lithium metal oxide cathode materials.

## Methods

### Materials

Iron acetylacetonate (Fe(acac)_3_, 99%), oleic acid (OAc, 90%), benzyl ether (BE, 99%) and nitrosonium tetrafluoroborate (NOBF_4_, 95%) were purchased from Sigma-Aldrich and used without any further purification. All solvents, including hexane, isopropanol, toluene and dimethylformamide (DMF), were in ACS reagent grade and were purchased from Fisher Scientific and used without further purification. Nanoparticle synthesis was performed using standard Schlenk techniques.

### Sample preparation

The Fe_3_O_4_ nanoparticles were synthesized by the decomposition of Fe(acac)_3_ in the presence of OAc, as modified from the approach in ref 5858. In a typical synthesis, 2 mmol of Fe(acac)_3_ was mixed with 1.28 ml of OAc and 10 ml of BE. The mixture was magnetically stirred and was kept under vacuum at 100 °C to generate a dark red solution. After evacuation at 100 °C for 1 h to remove all impurities and moisture, the system was filled with N_2_ and the solution was further heated to 290 °C at a rate of about 15 °C min^−1^ and kept at this temperature for 20 min. After cooling to room temperature, the NPs were separated by adding isopropanol, followed by centrifugation (8,500 r.p.m., 5 min). The NPs were further purified by sequential operations of dispersing in hexane, precipitation by adding isopropanol and centrifugation. The as-synthesized Fe_3_O_4_ nanoparticles were surface-passivated by OAc, which can be ligand-exchanged by NOBF_4_ according to literature^59^. About 0.1 g of NOBF_4_ was added into 10 ml DMF, resulting in immediately a light green solution. This NOBF_4_ DMF solution is added to the hexanes solution (10 ml) of Fe_3_O_4_ NPs (100 mg). The mixture was shaken for 10 min, followed by the addition of 25 ml of toluene. The NOBF_4_-modified Fe_3_O_4_ NPs were collected through centrifugation (7,000 r.p.m., 8 min). The Fe_3_O_4_ NPs after ligand exchange can be solubilized in DMF with sonication. To remove residual organics, the NPs were further purified by two cycles of re-dispersing in DMF and precipitation by adding toluene centrifugation. The NPs were either stored in DMF or evacuated at the room temperature overnight to make dry powder for further experiments.

### Electrochemical measurements

Composite electrodes were prepared with 80 wt% active material, 10 wt% polyvinylidene fluoride and 10 wt% acetylene carbon black in NMP (*N*-methyl-2-pyrrolidone) and cast onto copper current collectors. 2032-type coin cells were assembled in an argon-filled glove box using the composite electrode as the positive electrode and Li metal as the negative electrode. A Celgard separator 2400 and 1 M LiPF_6_ electrolyte solution in 1:1 w/w ethylene carbonate/dimethyl carbonate were used to fabricate coin cells. Battery testing was performed on computer controlled systems (Arbin BT2000 and BioLogic VMP3) at 21 °C.

### TEM characterization

The *in situ* TEM electrochemical cell was incorporated into a Nanofactory TEM-STM specimen holder, in which Fe_3_O_4_ nanoparticles dispersed onto a TEM half-grid with amorphous carbon support are analogous to the Fe_3_O_4_-C composite electrode, Li metal is coated onto a piezo-driven W probe as the counter electrode, with a thin layer of Li_2_O formed on Li metal as the solid electrolyte^36^,^37^,^38^. The Li and Fe_3_O_4_ were loaded onto the holder in an Ar-filled glove box and then transferred to TEM column using a sealed Ar bag to avoid air exposure. During the *in situ* TEM tests, a constant negative DC potential was applied to Fe_3_O_4_ electrode against the Li source during the lithiation process, and the lithiation processes were captured by real-time imaging in either TEM or STEM mode. We estimated the average discharge current density across the entire particle surface to be 0.017 mA cm^−2^. The *ex situ* samples after discharge in coin cells were examined accordingly. The *in situ* BF-STEM measurements were performed on a JEOL 2100F TEM operated at 200 kV. We have used a convergence angle of 12 mrad and a collection angle cutoff at ∼20 mrad. The high-resolution STEM imaging and analytical EELS were conducted on a Hitachi HD2700C STEM operated at 200 kV and equipped with a probe aberration corrector (spatial resolution <1 Å, energy resolution 0.35 eV).

### Theoretical calculations

The DFT calculations were performed using the Vienna *Ab initio* Simulation Package (VASP) within the projector augmented-wave approach with the Perdew−Burke−Ernzerhof generalized-gradient approximation. The DFT parameters were consistent with the parameters used in Materials Project (MP)^48^. A Hubbard *U* term of 5.3 eV is adopted for Fe^2+^ and Fe^3+^ in accordance with the MP and the previous testing^51^,^52^. The ground-state crystal structures of LiFe_3_O_4_ were obtained by enumerating 380 symmetrically distinctive configurations of Li/Fe disordering at the 16c sites using pymatgen^49^. Several magnetic orderings in ferromagnetic, anti-ferromagnetic and ferrimagnetic structures are tested for iron oxide compounds, such as Fe_3_O_4_, FeO, LiFe_3_O_4_ and LiFeO_2_, to identify the lowest energy state (more details provided in Supplementary Fig. 8 and Supplementary Table 1). The voltage plateaus are obtained using the DFT energies of all relevant compounds in the Li–Fe–O ternary space from the MP^50^.

The phase-field simulation was performed using the electrochemistry theory based on non-equilibrium thermodynamics developed by Bazant *et al*.^53^,^54^,^55^. The standard phenomenological model based on the Butler–Volmer equation^55^,^56^ and the Cahn–Hilliard equation^23^,^57^ was used for modelling the electrochemical kinetics. A homogeneous free-energy was comprised using piecewise functions of polynomial with continuity and differentiability at the segment points. The overpotential and strain energy are also considered in the simulation. More details are shown in Supplementary Figs 9–11, Supplementary Table 2 and Supplementary Methods.

## Additional information

**How to cite this article:** He, K. *et al*. Visualizing non-equilibrium lithiation of spinel oxide via *in situ* transmission electron microscopy. *Nat. Commun.* 7:11441 doi: 10.1038/ncomms11441 (2016).

## Supplementary Material

Supplementary Information Supplementary Figures 1-11, Supplementary Tables 1 & 2, Supplementary Methods and Supplementary References.

Supplementary Movie 1 In situ electron diffraction pattern during the entire lithiation process. The movie is accelerated by 100 times.

Supplementary Movie 2 In situ BF-STEM showing the two-step lithiation process via intercalation and conversion reactions. The movie is accelerated by 60 times.

Supplementary Movie 3 Lithium composition profile as a function of reaction time calculated by phase-field simulation.
