# Supplementary material for: Visualizing non-equilibrium lithiation of spinel oxide via in situ transmission electron microscopy
Source: Nat Commun. 2016 May 9;7:11441. doi: 10.1038/ncomms11441 (PMC4865808; doi:10.1038/ncomms11441)
Supplement: Supplementary Information — Supplementary Figures 1-11, Supplementary Tables 1 & 2, Supplementary Methods and Supplementary References. [file ncomms11441-s1.pdf]

Supplementary Figures

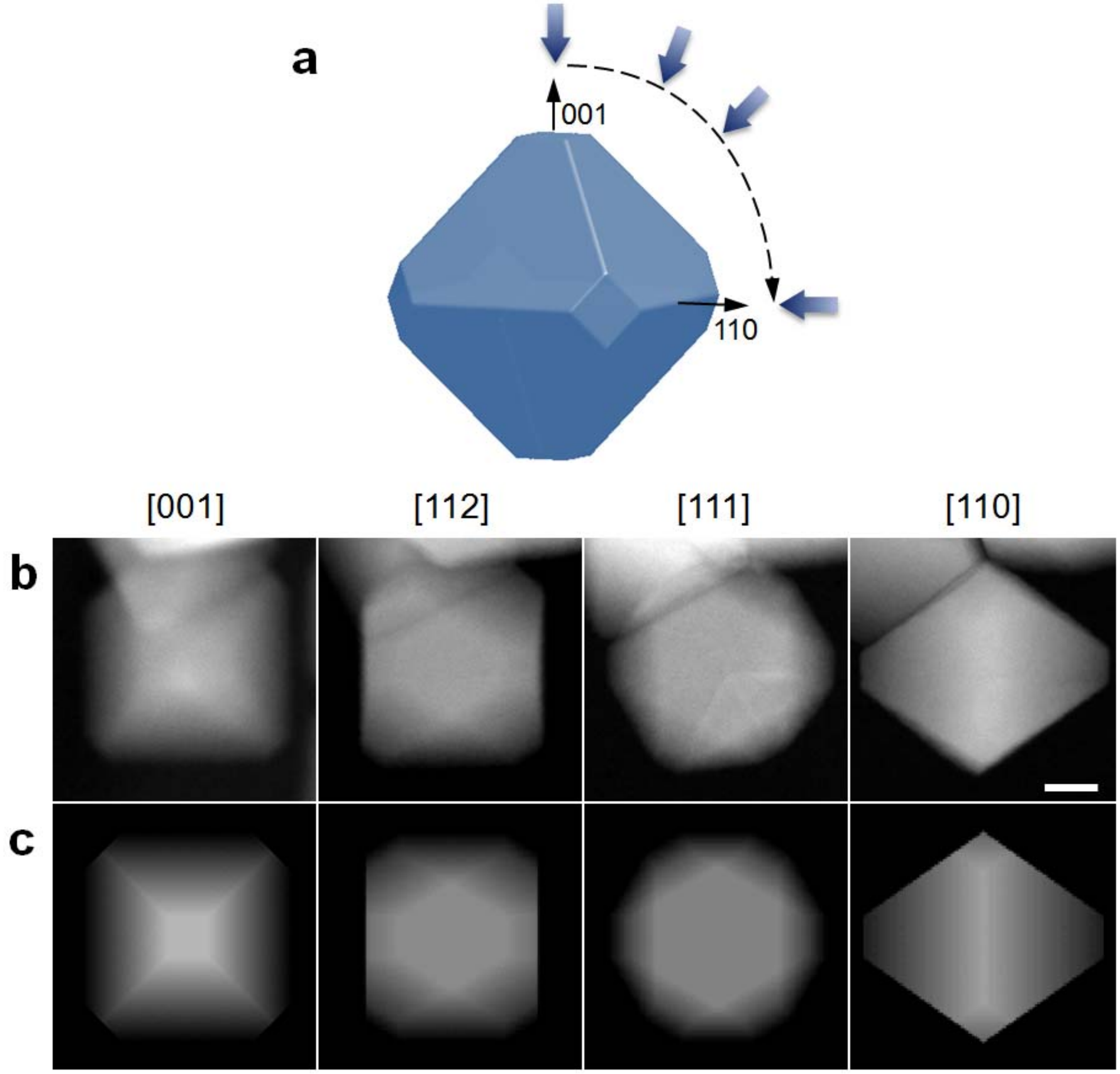

Supplementary Figure 1. (a) Schematic showing a model structure of a spinel Fe_3O_4 nanoparticle in shape of a truncated octahedron with exposed surfaces of {100} and {111} facets. Along specific crystal orientations, electron micrographs can show different characteristic projections. (b) Experimental and (c) simulated HAADF-STEM images showing the nanoparticle projection in square-like, rectangle-like, hexagon-like, and diamond-like shapes along [001], [112], [111], and [110] directions, respectively. Scale bar, 20 nm.

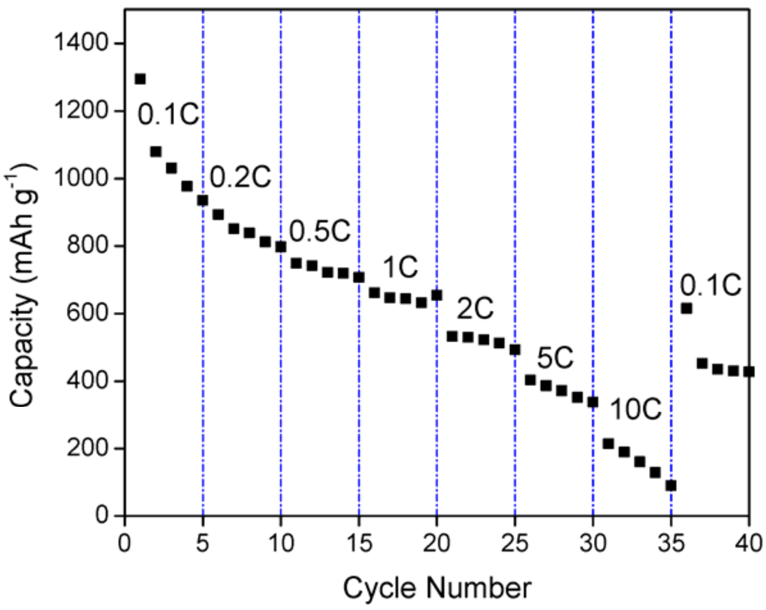

Supplementary Figure 2. The rate performance of Fe_3O_4 nanoparticle electrodes.

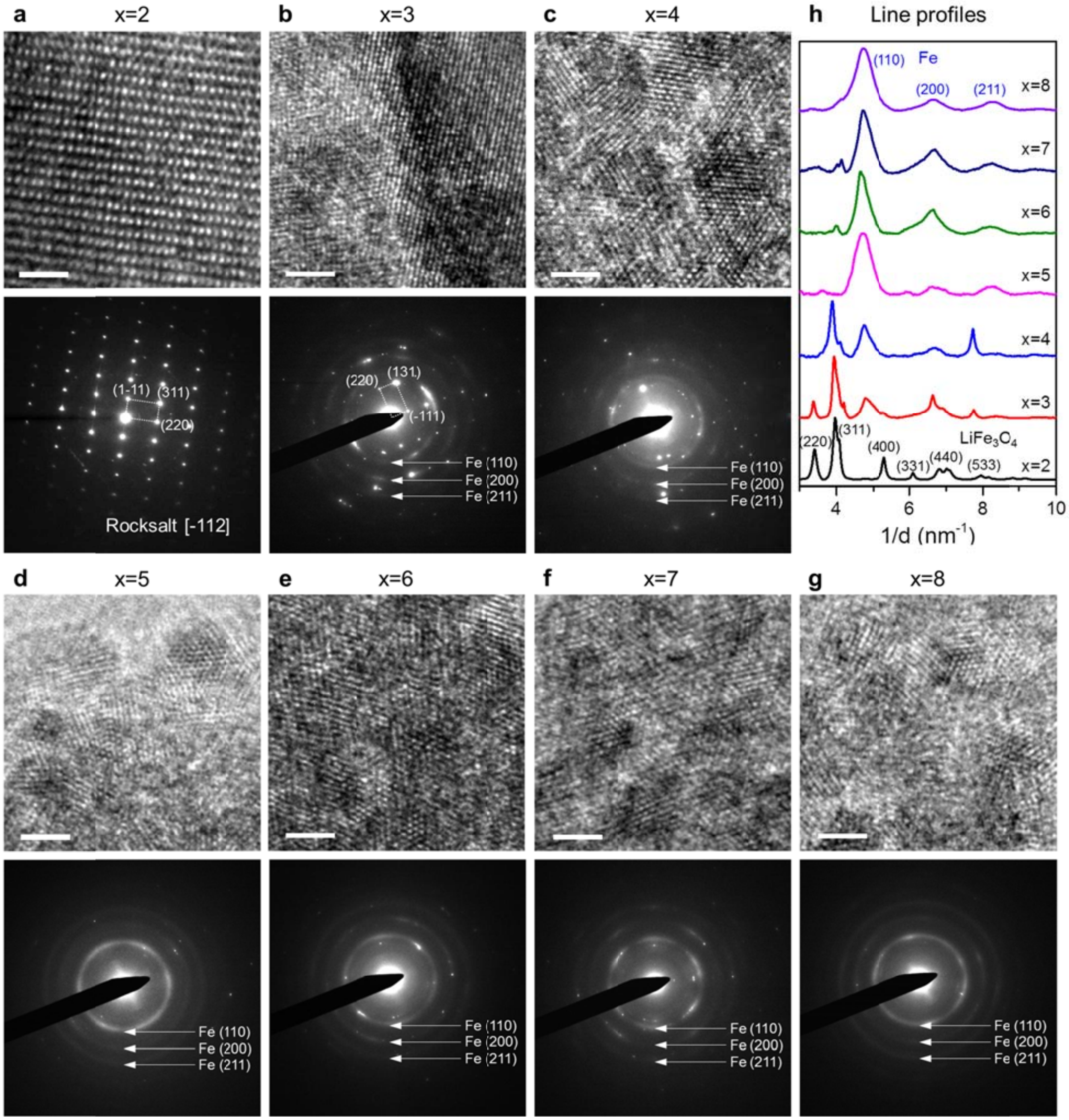

Supplementary Figure 3. (a-g) Representative HRTEM images and corresponding selected area diffraction patterns of the lithiated $Li_xFe_3O_4$ (x = 2, 3, 4, 5, 6, 7, 8). The diffraction spots in (b-g) are from the remaining $LiFe_3O_4$ rocksalt phase and diminish as lithiation approaching the end. (h) Radially averaged intensity line profiles of the electron diffraction in (a-g). Scale bars, 2 nm.

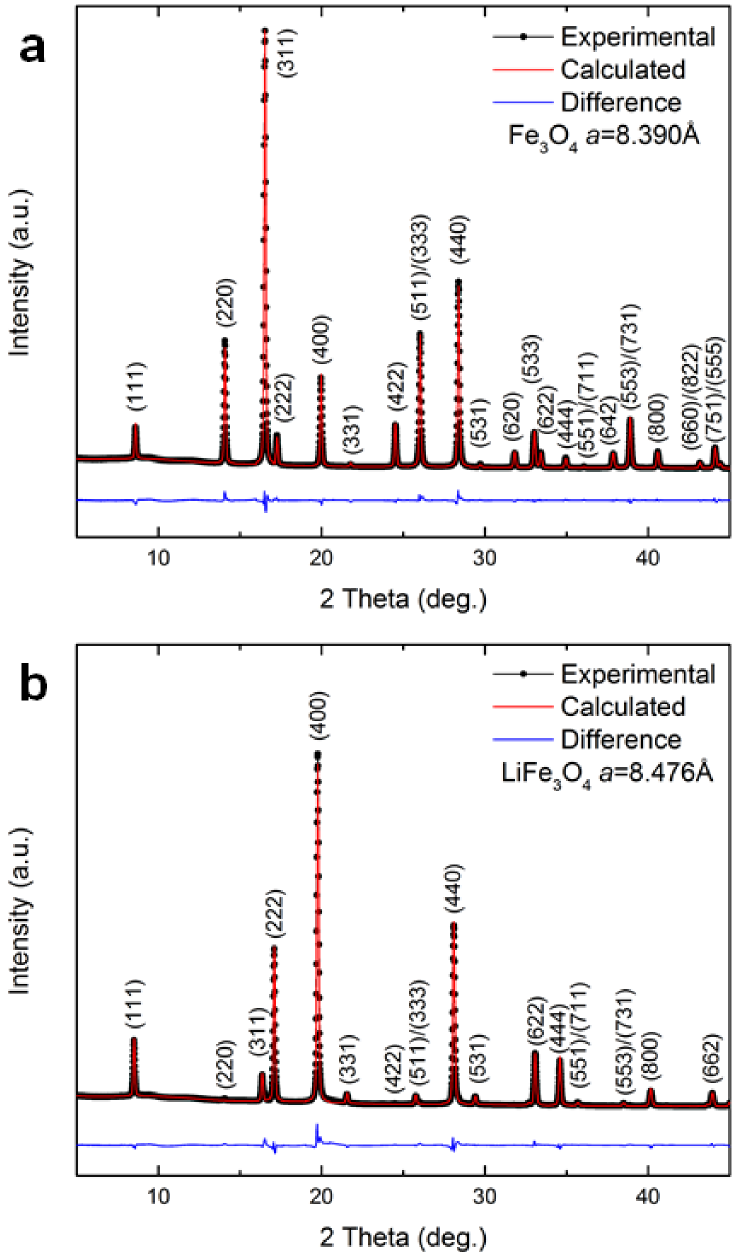

Supplementary Figure 4. Synchrotron XRD patterns of (a) pristine phase (x = 0) and (b) lithiated phases (x = 2). By fitting the experimental spectra using Rietveld refinement, the pristine phase is determined to be spinel structure with lattice parameter a = 8.390 Å, and the lithiated phase to be $LiFe_3O_4$ with lattice parameter a = 8.476 Å.

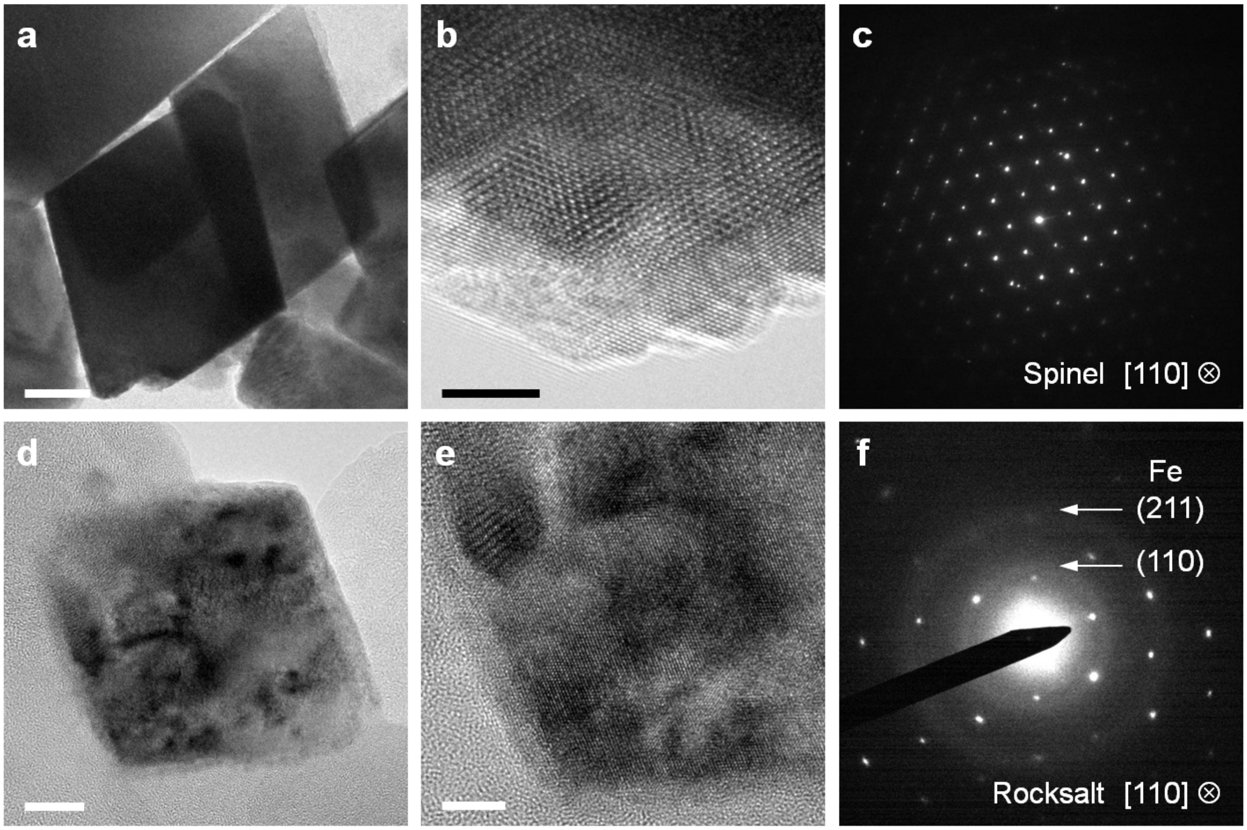

Supplementary Figure 5. (a) TEM and (b) HRTEM images, and (c) SAED pattern of the lithiated $Li_xFe_3O_4$ with x =2. The nanocrystal interior displays the spinel structure (as seen in SAED and HRTEM) with a portion on the surface showing the rocksalt structure (HRTEM). (d) TEM and (e) HRTEM images, and (f) SAED pattern of the lithiated $Li_xFe_3O_4$ with x =4. The entire nanocrystal displays the rocksalt structure (SAED reflection spots) along with the formation of a number of nanosize Fe particles (SAED diffraction rings). Fe nanoparticles can also be seen as darker contrast in TEM and HRTEM images. Scale bars, (a) 50nm; (b) 5nm; (d) 10 nm; (e) 5 nm.

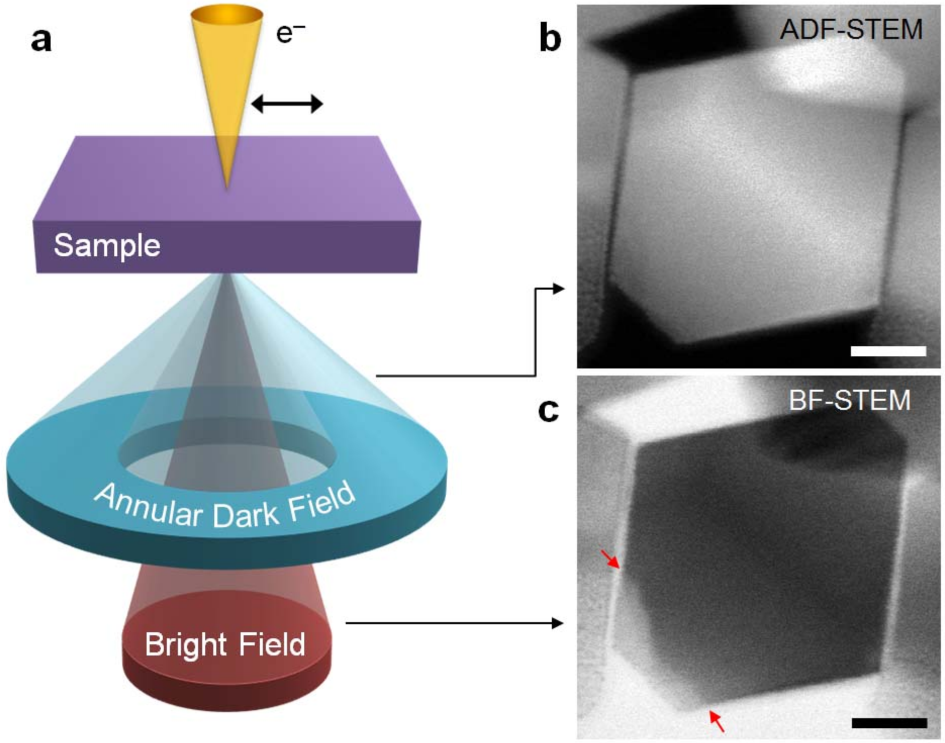

Supplementary Figure 6. (a) Schematics showing two different modes of STEM imaging by collecting either high-angle scattered electrons using an annular dark-field (ADF) detector, or low-angle scattered and directly transmitted electorns using a bright-field (BF) detector. The ADF-STEM image (b) contains highly scattered electron signals and gives a better sensitivity of Z (atomic number) contrast. The BF-STEM image (c) contains mostly coherent electron signals and is more sensitive to the diffraction contrast caused by local strains. Direct comparison between simultaneously acquired (b) ADF- and (c) BF-STEM images of the same sample region suggests that a better contrast of the lithiation front (indicated by red arrows) can be revealed by BF-STEM imaging. Scale bars, 20 nm.

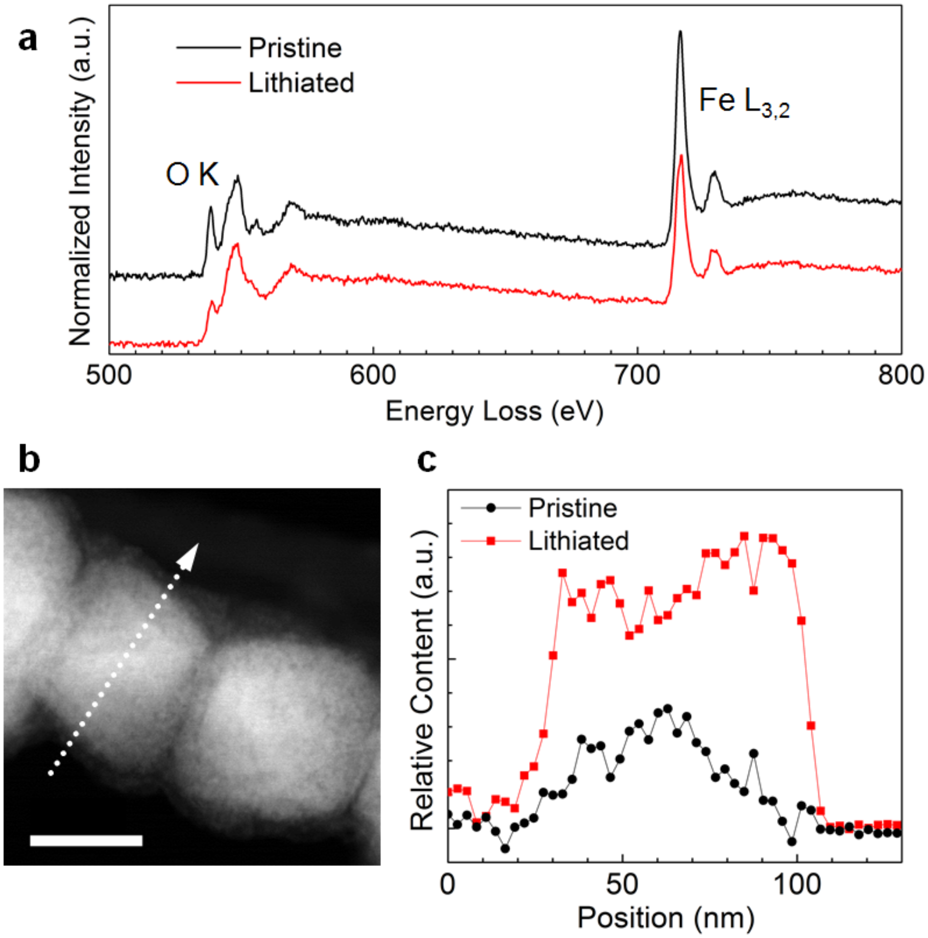

Supplementary Figure 7. (a) EELS spectra of pristine Fe_3O_4 and lithiated $Li_xFe_3O_4$ samples. (b) STEM image of half-lithiated nanoparticles. Scale bar, 50 nm. (c) Relative content of the pristine and lithated phases extracted from the EELS linescan along the arrow in (b).

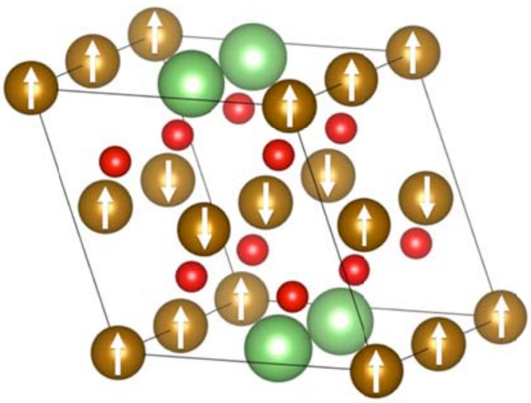

Supplementary Figure 8. The ground-state magnetic ordering of $LiFe_3O_4$ (Green: Li, Red: O, Gold: Fe).

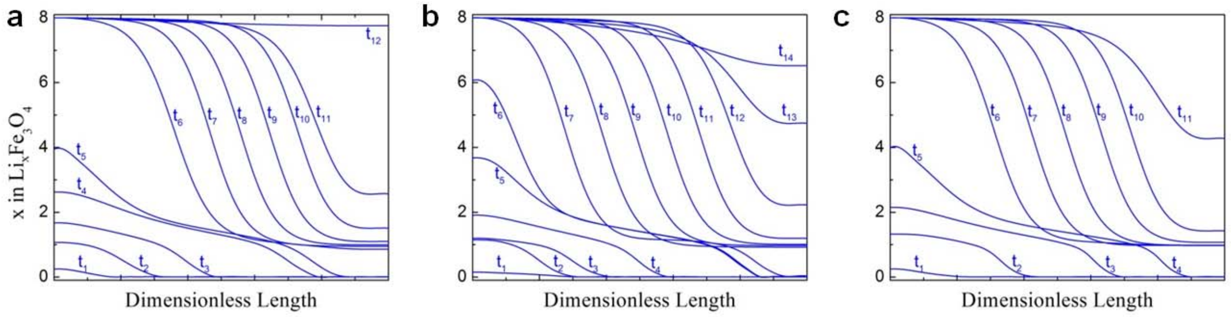

Supplementary Figure 9. Time-sequenced Li composition profile with different b_1 values. (a) b_1 = 200; (b) b_1 = 400; and (c) b_1=800. Other parameters remain unchanged.

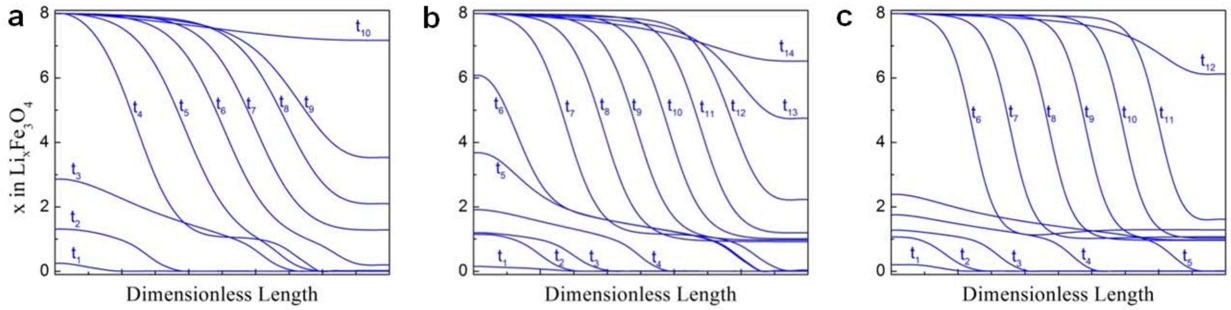

Supplementary Figure 10. Time-sequenced Li composition profile with different b_2 values. (a) b_2 = 2.5; (b) b_2 =5; (c) b_2 = 10. Other parameters remain unchanged.

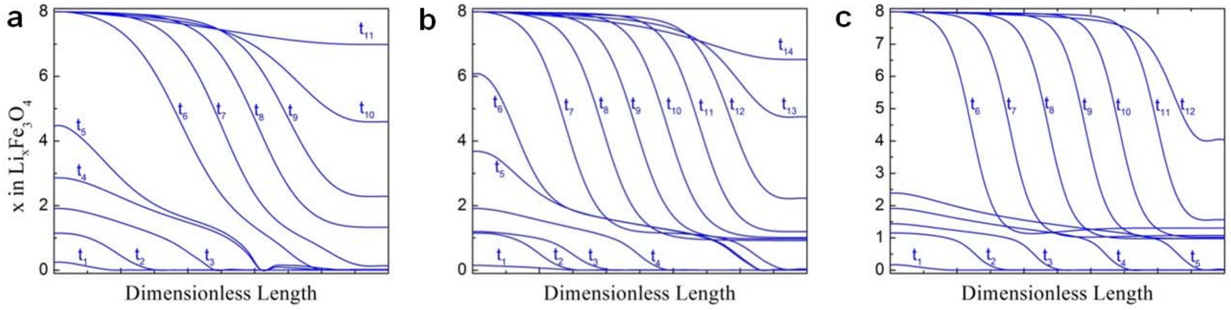

Supplementary Figure 11. Time-sequenced Li composition profile with different combinations of b_1 and b_2. (a) b_1 = 200, b_2 = 2.5; (b) b_1 = 400, b_2 = 5; (c) b_1=800, b_2=10. Other parameters remain unchanged.

Supplementary Tables

Supplementary Table 1. The ground-state magnetic ordering of the iron oxide phases. Other phases show ferromagnetism in agreement with the *Materials Project*.

Compound	Magnetic type with the lowest energy	Magnetic moment (μB/f.u.)
Fe_3O_4	Ferrimagnetic	4.00
FeO	Antiferromagnetic	0.00
$LiFeO_2$	Antiferromagnetic	0.00
$LiFe_3O_4$	Antiferromagnetic	0.00

Supplementary Table 2. Parameters used in phase-field simulations.

Parameter	Value
b_1	400
b_2	5
α	0.5
κ	0.5
L	100
I	0.001
$\widetilde{M}$	10

Supplementary Methods

Phase-Field Modeling

Our *in situ* experiments and DFT calculations have confirmed that the Fe_3O_4 nanocrystals undergo two reaction stages during the lithiation process, *i.e.*, from original Li-free Fe_3O_4 to Li-intercalated $LiFe_3O_4$ phase, and then to a composite of Li_2O+Fe. Here, we explain the evolution process using the electrochemistry theory based on non-equilibrium thermodynamics developed by Bazant and coworkers.[1, 2] The standard phenomenological model of electrode kinetics is the Butler-Volmer equation[1-3]

$$\frac{\partial c}{\partial t} = I_0\left[\exp\left(-\frac{\alpha ne\eta}{k_B T}\right) - \exp\left(\frac{(1-\alpha)ne\eta}{k_B T}\right)\right] \tag{1}$$

where $\frac{\partial c}{\partial t}$ is the change rate of the local filling fraction c of Li-ion; α, the electron-transfer symmetry factor, is approximately constant for many reaction; I_0 is the exchange current; η is activation overpotential; ne is the net charge transferred from the solution to the electrode; k_B is Boltzmann's constant and T is temperature. The exchange current will be written as[1]

$$I_0 = K_0(1-\tilde{c})^s \exp\left[\tilde{\sigma} : \Delta\varepsilon + \alpha\frac{\delta\tilde{G}}{\delta\tilde{c}}\right] \tag{2}$$

$$K_0 = kc_s\left(a_+ a_e^n\right)^{1-\alpha} \exp\left(-\frac{\lambda_0}{4k_B T}\right) \tag{3}$$

where k is the insertion/extraction rate; c_s is the reaction site density; a_+ and a_e is respectively the ionic activity in the electrolyte and the electron activity; λ_0 is the reorganization energy. The letters with tilde in Eq. (2) represent these variables are scaled to dimensionless form. For example, $\tilde{c} = \frac{c}{c_s}$, $\tilde{\sigma} = \frac{\sigma}{c_s k_B T}$, $\tilde{G} = \frac{G}{k_B T}$. In Eq. (2), $\Delta\varepsilon$ is the activation strain; $\frac{\delta\tilde{G}}{\delta\tilde{c}}$ is the functional derivative of Gibbs free energy functional. Based on the Cahn-Hilliard phase field model,[3]

$$\frac{\delta\tilde{G}}{\delta\tilde{c}} = \frac{\partial\tilde{f}}{\partial\tilde{c}} - \tilde{\kappa}\tilde{\nabla}^2\tilde{c} \tag{4}$$

where $\tilde{f} = \frac{f}{k_B T}$ is the homogeneous free energy function; $\tilde{\kappa} = \frac{\kappa}{L^2 c_s k_B T}$ is the Cahn-Hilliard gradient energy coefficient; and $\tilde{\nabla} = L\nabla$ (L is the length scale of sample). The activation overpotential in Eq. (1) can be written as

$$\tilde{\eta} = \frac{\eta}{k_B T} = \frac{\Delta\tilde{\mu}}{ne} \tag{5}$$

$\Delta\tilde{\mu} = \dfrac{\Delta\mu}{k_B T}$ is the thermodynamic driving force. It is the consistent definition of diffusional chemical potential. For an inhomogeneous system, the simplest approximation is $\Delta\tilde{\mu} = \dfrac{\delta\tilde{G}}{\delta\tilde{c}}$. For Li-ion battery electrodes, the overpotential is

$$\tilde{\eta} = \frac{1}{ne}\frac{\delta\tilde{G}}{\delta\tilde{c}} + \Delta\tilde{\Phi} + \tilde{\sigma} : \varepsilon \tag{6}$$

$\Delta\tilde{\Phi} = \dfrac{ne\Delta\Phi}{k_B T}$ is the local voltage drop across the interface; and $\tilde{\sigma} : \varepsilon$ is strain energy due to Li-ion insertion. The stress $\tilde{\sigma} = \dfrac{\sigma}{c_s k_B T}$ and the strain ε are resulted from lattice distortion with Li-ion insertion. Obviously, the strain depends on Li-ion concentration. For simplicity, we usually assume the strain linearly depends on Li-ion concentration. Therefore, the strain energy due to Li-ion insertion is quadratic function of Li-ion concentration. Appling the above theory to Li-ion battery electrodes, a_+ and a_e are constants and $\Delta\varepsilon = 0$ in the electrolyte,[1] and substituting Eq. (2) and Eq. (6) into Eq. (1), we have

$$\frac{\partial\tilde{c}}{\partial\tilde{t}} = \tilde{I}_0\left[\exp(-\alpha\tilde{\eta}) - \exp((1-\alpha)\tilde{\eta})\right] \tag{7}$$

where $\tilde{t} = k\left(a_+ a_e^n\right)^{1-\alpha} \exp\left(-\dfrac{\lambda_0}{4k_B T}\right)t$; $\tilde{I}_0 = (1-\tilde{c})^s \exp\left(\alpha\dfrac{\delta\tilde{G}}{\delta\tilde{c}}\right)$. The total current integrated over the active facet

$$\tilde{I}(\tilde{t}) = \int_{\tilde{A}} \frac{\partial\tilde{c}}{\partial\tilde{t}} d\tilde{x}d\tilde{y} \tag{8}$$

is controlled while solving for $\Delta\tilde{\Phi}$. $\tilde{A}$ in Eq. (8) is the dimensionless surface area of the active facet. The Li-ion concentration at the interface of electrode and electrolyte can be determined by Eq. (1) - (8). Inside the electrode, the evolution of Li-ion concentration is governed by the Cahn-Hilliard equation[4, 5]

$$\frac{\partial\tilde{c}}{\partial\tilde{t}} = \nabla\tilde{M}\tilde{c}\nabla(\Delta\tilde{\mu}) \tag{9}$$

where $\tilde{M}$ is the Li mobility tensor, which is in general a function of Li-ion concentration. It is assumed to be a constant in our simulations.

In order to describe the chemical kinetics using the non-equilibrium thermodynamics, we need a homogeneous free energy with three local minima. The three minima correspond to Fe_3O_4, $LiFe_3O_4$, and Li_2O+Fe, respectively. We use piecewise functions of polynomial to construct the homogeneous free energy. The piecewise functions are written as

$$f(c) = \begin{cases} b_1 c^2 (a_1 - c)^2 & c < a_1 \\ b_2 (a_1 - c)^2 (a_2 - c)^2 & c \geq a_1 \end{cases} \tag{10}$$

The three local minima are located at $c = 0$, $c = a_1$, and $c = a_2$, respectively. The coefficients b_1 and b_2 determine the maximum (energy barrier) between $c = 0$, and $c = a_1$, and $c = a_1$, and $c = a_2$. The piecewise functions of Eq. (10) are still continuous and differentiable at the point $c = a_1$ between the pieces. Therefore, we can assure the free energy and chemical potential of system is continuous. For our case, we consider the concentrations of three phases at $c = 0$, $a_1 = 0.125$ and $a_2 = 1$, equivalent to Li composition $x = 0$, 1, and 8, respectively. We change the coefficients b_1 and b_2 to modulate the heights of energy barriers.

Under galvanostatic conditions, Eq. (8) is an integral constraint the implicitly determines the voltage $\Delta\widetilde{\Phi}$. Eqs. (1) - (8) describe the electrochemical phase transformation kinetics. It provides a simple paradigm to understand non-equilibrium pattern formation driven by an applied voltage or current.[1-2] Solving Eqs. (1) - (8), the change of voltage with average composition and the evolution of Li-ion concentration with time can be obtained. We employed the explicit finite-difference method to numerically solve Eqs. (1) - (8), adjusting $\Delta\widetilde{\Phi}$ at each time interval $\Delta t = 10^{-3}$ to maintain the constant current. In the model, we assume that the concentration of lithium ions at the surface of sample keeps a constant, and then decreases rapidly with the gradual in-depth internal materials in initial time $\widetilde{t} = 0$. A half-Gaussian distribution (the first line in Figure 5d) is used to describe the initial concentration distribution. The range of strain energy is about 10^{-4} - 10^{-2} eV with strain 0.01 - 0.1. The pair interaction energy is about $2k_BT_c \approx 0.1$ eV at critical temperature $T_c = 600$ K of phase separation.[1] It implies that the strain energy with small strain less influence on phase separation. However, large strain will suppress phase separation and lead to non-crystalline formation.[5]

Many parameters can affect the simulations in the phase-field model. Singh *et al.*[6] and Bai *et al.*[2] have discussed the effects of applied voltage, gradient energy coefficient and applied current. The parameters used in our simulations have referred to these studies. The barrier of free energy is on the same order of magnitude as the free energy function used by Bai *et al.*[2] The specific parameters are listed in Supplementary Table 2. In order to initiate the phase transformation, 1% perturbation of Li-ion concentration is induced in each cycle.

The results of phase-field simulation are shown in Figure 5c, d. Figure 5c shows the calculated discharge voltage profile, which records the voltage responses with average lithium concentration at a constant current. Figure 5d shows the Li composition profile as a function of reaction time. The time stamps of t_1 - t_{14} use the uniform interval. Two reaction steps are found during the entire lithiation: first, the rocksalt $LiFe_3O_4$ phase forms and undergoes a rapid growth (t_1 - t_4); meanwhile, the conversion reaction also occurs but at a much slow speed (t_5 - t_7); afterward, the conversion phase quickly expands (t_8 - t_{14}) and have the sample fully lithiated.

In our modeling, a new form of free energy function, as expressed in Eq. (10), is introduced for describing the lithiation process in Fe_3O_4. Therefore, we need to figure out the effects of the parameters b_1 and b_2 in Eq. (10) on the electrochemical phase transformation kinetics. We performed simulations by varying b_1 and b_2 individually, or changing both of them proportionally. Supplementary Figure 9-11 shows a series of time-sequenced Li composition profiles with the change of b_1 and/or b_2. Comparing these figures, we found that the smaller b_1

and larger b_2 favors the growth of $LiFe_3O_4$ phase; and similarly, smaller b_2 and larger b_1 promotes the speed of the conversion reaction. It is straightforward to understand since b_1 and b_2 determine the energy barrier between Fe_3O_4 and $LiFe_3O_4$, and between $LiFe_3O_4$ and Li_2O+Fe, respectively. We also noted that b_1 and b_2 will influence the interfacial structure even with the Cahn-Hilliard gradient energy coefficient unchanged. For example, smaller b_1 and b_2 values will lead to larger interfacial widths. It is consistent with the effect that we found in previous calculations.[7] In addition to b_1 and b_2, other variables, such as the Cahn-Hilliard gradient energy coefficient, Li^+ mobility in electrode, and applied current, may also affect the evolution of lithiation process. Nevertheless, despite the minor effect on reaction speeds and interfacial structures, the intrinsic nature the two-step lithiation has been successfully reproduced.

It is worth noting that Eq. (10) is an empirical formula. Some experimental phenomena, such as Li composition profiles with respect to time, evolution of phase transition, can be qualitatively described using the phase-field theory. In order to describe the experimental results of a specific material more quantitatively and accurately, the parameters of b_1 and b_2, the Cahn-Hilliard gradient energy coefficient, and Li^+ diffusion coefficient, need to be determined by relevant experimental measurements.

Supplementary References

1. Bazant, N. Z. Theory of Chemical Kinetics and Charge Transfer based on Nonequilibrium Thermodynamics. *Acc. Chem. Res*. **46**, 1144-1160 (2013).

2. Bai, P., Cogswell, D. A. & Bazant, M. Z. Suppression of Phase Separation in $LiFePO_4$ Nanoparticles During Battery Discharge. *Nano Lett*. **11**, 4890-4896 (2011).

3. Newman, J. *Electrochemical Systems, 2nd Ed*. (Prentice Hall, 1991).

4. Cahn, J. W. & Hilliard, J. E. Free Energy of a Nonuniform System. I. Interfacial Free Energy. *J. Chem. Phys.* **28**, 258-267 (1958).

5. Tang, M.; Carter, W. C. & Chiang, Y. M. Electrochemically Driven Phase Transition in Insertion Electrodes for Lithium-Ion Batteries: Examples in Lithium Metal Phosphate Olivines, *Annu. Rev. Mater. Res.* **40**, 501-529 (2010).

6. Singh, G. K.; Ceder, G. & Bazant, M. Z. Intercalation dynamics in rechargeable battery materials: General theory and phase-transformation waves in $LiFePO_4$, *Electrochim. Acta* **53**, 7599-7613 (2008).

7. Meng, Q. & Zhu, Y. Strustural modification of twin boundaries in $YBa_2Cu_3O_6+\eta$ oxides: Effects of oxygen concentration and temperature. *Phys. Rev. B* **75**, 174501 (2007).
